# Mechanism of Resveratrol in Alleviating Ochratoxin A-Induced Damage to Porcine Ovarian Granulosa Cells

**DOI:** 10.3390/antiox15101339

**Published:** 2026-10-16

**Authors:** Li-Yan Hou, Ying Zhou, Ya-Xuan Li, Shu-Hao Fan, Zong-Jun Yin

**Affiliations:** College of Animal Science and Technology, Anhui Agricultural University, Hefei 230036, China

**Keywords:** ochratoxin A, inflammation, resveratrol, porcine ovarian granulosa cells, NF-κB/HIF-1 signaling pathway

## Abstract

Resveratrol (RES) is a natural polyphenolic compound that protects the organism against oxidative stress and various metabolic injuries. Ochratoxin A (OTA) is a widely occurring mycotoxin with established reproductive toxicity in monogastric animals, including pigs. Nevertheless, its molecular mechanisms remain elusive. This study aimed to investigate OTA-induced cellular injury and the protective role of RES using porcine granulosa cells as an in vitro model. OTA treatment significantly reduced cell viability, increased lactate dehydrogenase release, induced oxidative damage, and promoted apoptosis. Transcriptomic analysis revealed that OTA markedly activated the NF-κB inflammatory pathway while suppressing the expression of HIF-1 signaling-related genes. Consistently, RT-qPCR showed that OTA upregulated the mRNA levels of *TNF-α*, *p50*, *p65*, *IL-6*, and *CXCL-14*. Furthermore, ELISA demonstrated that OTA increased the protein secretion of IL-1β, IL-8, TNF-α, MCP-1, and IL-6. Western blot analysis indicated that OTA decreased IκBα protein levels but did not alter p65 expression. Notably, RES co-treatment effectively reversed all the above-mentioned alterations and restored the aberrant expression of NF-κB and HIF-1 pathway genes to levels close to those of the control group. In conclusion, RES alleviates OTA-induced damage in porcine ovarian granulosa cells via modulating oxidative stress, inflammation response and apoptosis.

## 1. Introduction

More than 200 fungal species can produce mycotoxins (e.g., aflatoxins, zearalenone, ochratoxin A, fumonisins, and trichothecenes such as deoxynivalenol and T-2 toxin) [1,2], and these toxins can significantly affect the health and reproductive performance of livestock. Ochratoxin A (OTA) is an important mycotoxin produced by certain species of Aspergillus (e.g., Aspergillus ochraceus) and Penicillium, with a wide range of contamination, and is commonly found in cereals (corn, wheat, oats, etc.), nuts, fruits and their processed products (raisins, coffee, wine), as well as in animal feed [3,4,5]. Accumulating evidence indicates that OTA accumulation through the food chain induces nephrotoxicity, hepatotoxicity, and reproductive toxicity in both animals and humans [6,7]. Upon entry into the body, OTA readily binds to plasma proteins and predominantly accumulates in kidney tissues [8], where it elicits tubular atrophy, interstitial fibrosis, and other nephrotoxic lesions. In hepatocytes, OTA triggers oxidative stress and apoptosis via the aryl hydrocarbon receptor (AhR) signaling pathway, while cells simultaneously activate the Nrf2 pathway to synthesize phase II detoxifying enzymes as a defensive response [9,10]. Moreover, OTA exposure provokes oxidative stress and activates apoptotic pathways in human embryonic stem cells, thereby interfering with cell proliferation and differentiation [11]. In male rats, chronic low-dose OTA exposure impairs testicular function and reduces semen quality, and this reproductive toxicity is exacerbated by concurrent alcohol consumption [12]. Additionally, low-dose OTA decreases the ability of ovine oocytes to block polyspermy and significantly increases the blastocyst apoptosis rate [13].

Porcine ovarian granulosa cells play a pivotal role in regulating follicular development and the secretion of reproductive hormones. they directly determine ovulation number and oocyte quality in sows, thereby affecting sow fertility and the economic returns of swine production [14,15]. However, the precise toxic mechanisms and underlying causes of OTA-induced damage to porcine ovarian granulosa cells remain largely unclear. Furthermore, no effective intervention is currently available to mitigate the cytotoxicity of OTA.

In recent years, RES, a natural polyphenolic compound that is widely distributed in grapes, berries, and peanuts and that has the advantage of low extraction cost, has garnered considerable attention [16,17]. Studies have demonstrated that RES directly scavenges reactive oxygen species, inhibits lipid peroxidation, and enhances endogenous antioxidant enzyme activities, thereby exerting cytoprotective effects [18,19]. Moreover, it has been shown to reduce neuroinflammatory responses, suppress microglial activation, and attenuate ROS-induced neurotoxicity, thus alleviating neurological disorders including Alzheimer’s disease and Parkinson’s disease [20,21]. Relevant studies have shown that RES attenuates H_2_O_2_-induced apoptosis in human ovarian granulosa cells [22,23] and alleviates H_2_O_2_-induced apoptosis and oxidative stress in rat luteinized granulosa cells [24]. RES attenuates apoptosis and oxidative stress in oocytes induced by zearalenone and doxorubicin [25,26]. During spermatogenesis, RES enhances SIRT1 activity and modulates FOXO, p53, and MAPK-associated signaling cascades, which in turn promotes the antioxidant capacity and DNA repair efficiency of spermatogenic cells [27,28]. Furthermore, its anti-inflammatory properties, mediated by inhibition of the NF-κB pathway, lower the expression of pro-inflammatory cytokines, consequently ameliorating the detrimental effects of an unfavorable uterine microenvironment on embryonic development [29]. Based on transcriptomic analysis, this study aimed to investigate the toxic effects of OTA on porcine ovarian granulosa cells and elucidate the underlying molecular mechanisms. Furthermore, we evaluated whether RES supplementation could attenuate OTA-induced cellular injury, providing a potential protective strategy against mycotoxin-induced reproductive toxicity.

## 2. Materials and Methods

### 2.1. Cell Source and Ethical Statement

Porcine ovarian granulosa cells were purchased from Zhien Biotech (Hefei, China). No animal ethics approval was required as the cells were derived from slaughterhouse materials.

### 2.2. Reagents and Antibodies

OTA (Cat# HY-N6788) and RES (Cat# HY-16561) were purchased from MedChemExpress (MCE, Monmouth Junction, NJ, USA). Dimethyl sulfoxide (DMSO, cell culture grade, Cat# D8371) was obtained from Solarbio (Beijing, China). OTA and RES powders were dissolved in DMSO to obtain 10 mM stock solutions. For cell interventions, stock solutions were serially diluted with complete culture medium to achieve the required working concentrations. The final volume fraction of DMSO in all culture wells was maintained ≤0.1% (*v*/*v*), and vehicle control groups were treated with an identical concentration of DMSO. DMEM/F12 medium and 0.25% EDTA-trypsin (Cat# 25200056) were purchased from Gibco (Grand Island, NY, USA). Penicillin–streptomycin solution (100×, Cat# BL505A) and 1× PBS buffer (Cat# BL302A) were obtained from Biosharp (Beijing, China). Fetal bovine serum (FBS, Cat# 03.C16001DC) was purchased from EallBio (Beijing, China). The Cell Counting Kit-8 (CCK-8, Cat# E606335) was obtained from Sangon Biotech (Shanghai, China). Assay kits for superoxide dismutase (SOD, Cat# BC5165), malondialdehyde (MDA, Cat# BC0025), glutathione (GSH, Cat# BC1175), and lactate dehydrogenase (LDH, Cat# BC0685) were purchased from Solarbio (Beijing, China). The Annexin V-FITC/PI apoptosis detection kit (Cat# C1075S) was obtained from Beyotime (Shanghai, China). The reactive oxygen species (ROS) assay kit (Cat# E004-1-1) was purchased from Nanjing Jiancheng Bioengineering Institute (Nanjing, China).

### 2.3. Cell Culture and Treatment

The cells were cultured in DMEM/F-12 (1:1) medium supplemented with 10% FBS and 1% antibiotics (10 μg/mL streptomycin and 10,000 IU/mL penicillin). The cultures were maintained at 37 °C in a humidified atmosphere containing 5% CO_2_. The culture medium was renewed every 48 h. After reaching approximately 80% confluence, cells were passaged or subjected to the indicated treatments.

### 2.4. CCK-8 Assay

Cell viability was evaluated using the CCK-8 kit. To assess the effects of ochratoxin A (OTA) and resveratrol (RES) on the viability of porcine ovarian granulosa cells, cells were seeded into 96-well plates at a density of 5 × 10^3^ cells per well. After 12 h of incubation at 37 °C in a 5% CO_2_ atmosphere to allow attachment, the old medium was aspirated, and the cells were gently washed once with pre-warmed PBS to remove non-adherent cells. The cells were then treated with fresh medium containing various concentrations of OTA (0, 1, 2, 4, 8, 16, 32, 64 µM) or RES (0, 0.5, 1, 2, 4, 8, 10, 15, 20 µM) for 24 h at 37 °C. After treatment, the medium was removed, and the cells were washed twice with PBS. Subsequently, 100 µL of fresh complete growth medium containing 10 µL of CCK-8 solution was added to each well, followed by incubation for 3 h at 37 °C in a 5% CO_2_ atmosphere. The absorbance was measured at 450 nm using a microplate reader (Nanjing Detie, Nanjing, China, model HBS-1096A). Each treatment was performed in quadruplicate, and cell viability was calculated based on the absorbance values. The assay was performed as previously described [30,31].

### 2.5. Detection of LDH Release in Cell Culture Medium

Porcine ovarian granulosa cells were seeded into 96-well plates at a density of 5 × 10^3^ cells per well and cultured for 12 h. Based on the CCK-8 results, subsequent treatments were performed with OTA at 16 μM and RES at 15 μM. The cells were treated with the respective concentrations of the agents and further incubated for 48 h. The culture medium was then collected and centrifuged at 400× *g* for 5 min at 4 °C. The supernatant was used to measure LDH activity using an L-LDH assay kit (Solarbio, Beijing, China), and the absorbance was measured at 450 nm using a microplate reader. The supernatant was used without dilution according to the manufacturer’s protocol. Each treatment was performed in triplicate.

### 2.6. Measurement of Reactive Oxygen Species and Antioxidant Indicators

Round glass coverslips (20 mm in diameter) were placed into 12-well plates, and porcine ovarian granulosa cells were seeded into the plates and cultured for 24 h. The cells were then treated with OTA, RES, or the combination of OTA and RES for 48 h. After three washes with DMEM, the cells were incubated with 1 mL of diluted DCFH-DA (10 μM) for 30 min at 37 °C in the dark. After incubation, the cells were washed twice with PBS to remove excess probe, and ROS levels were observed under a fluorescence microscope with excitation at 488 nm and emission at 525 nm.

To evaluate the effect of OTA exposure on the antioxidant capacity of porcine ovarian granulosa cells and the protective effect of RES, the cells were divided into four groups: control group, OTA-treated group, RES-treated group, and OTA+RES co-treated group. Following the respective treatments, the cells were collected and lysed. After centrifugation at 12,000× *g* for 10 min at 4 °C, the supernatant was collected and used without further dilution for the measurement of MDA content, SOD activity, and GSH content using corresponding assay kits (Solarbio, Beijing, China) according to the manufacturer’s instructions. The absorbance was measured at 532 nm and 600 nm (the difference between the two readings, after blank subtraction, was used for quantification) for MDA, 450 nm for SOD, and 412 nm for GSH using a microplate reader. All experiments were carried out in at least three independent biological replicates. Meanwhile, each biological replicate contained three wells as technical replicates in this experiment, and the final values were calculated as the mean of the three technical replicates to ensure the reliability of our data.

### 2.7. Apoptosis Detection

Apoptosis and necrosis were assessed using the YO-PRO-1/PI Apoptosis and Necrosis Detection Kit (Beyotime, Shanghai, China), which is a dual-fluorescence assay based on the DNA-binding green fluorescent dye YO-PRO-1 (YP1) and the red fluorescent dye propidium iodide (PI), according to the manufacturer’s instructions. Porcine ovarian granulosa cells were seeded into 96-well plates and cultured for 12 h at 37 °C in a 5% CO_2_ atmosphere. The cells were then divided into four groups: control group, 16 μM OTA group, 15 μM RES group, and 16 μM OTA + 15 μM RES co-treatment group. After 24 h of treatment, the culture medium was removed, and the cells were washed once with PBS. Subsequently, 100 μL of YO-PRO-1/PI detection working solution was added to each well, and the plates were incubated for 10 min at 37 °C in the dark. Following incubation, fluorescence staining was observed under a fluorescence microscope, and all procedures were performed under light-protected conditions.

### 2.8. Real-Time Quantitative PCR

Porcine ovarian granulosa cells were cultured in 12-well plates. Total RNA was extracted using an RNA isolation kit (Vazyme, Nanjing, China) according to the manufacturer’s instructions. After RNA extraction, reverse transcription was performed using the PrimeScript RT Reagent Kit with gDNA Eraser (Takara, Shiga, Japan). Specifically, genomic DNA was removed from each sample as per the kit protocol, followed by the preparation of a 20 μL reverse transcription reaction mixture. The reaction was carried out at 37 °C for 15 min and terminated at 85 °C for 5 s. The resulting product was used as the cDNA template for subsequent real-time quantitative PCR. RT-qPCR was performed using TB Green^®^ Premix ExTaq™ II (TB stands for TaqMan Binding; Takara, Shiga, Japan). The primer sequences for the target genes are listed in Table S1. Relative gene expression levels were calculated using the 2^−ΔΔCt^ method.

### 2.9. Western Blot

Porcine ovarian granulosa cells were cultured in 6-well plates and treated according to the experimental design. After treatment, the cells were washed three times with PBS, each for 5 min. Total protein was then extracted, and the protein concentration was determined using a BCA Protein Assay Kit (Beyotime, Shanghai, China) following the manufacturer’s instructions. Protein samples were mixed with loading buffer and subjected to Western blotting. Following electrophoresis, the separated proteins were transferred onto a PVDF membrane. The membrane was blocked with 5% BSA for 2 h and then incubated with primary antibodies overnight at 4 °C. On the next day, the primary antibodies were removed, and the membrane was washed three times with TBST, followed by incubation with the corresponding secondary antibodies for 1 h at room temperature. Finally, protein expression was detected using an ECL chemiluminescent substrate and a gel imaging system. Antibodies used for Western blotting were as follows: anti-GAPDH (LF205, EpiZyme, Shanghai, China), anti-NFKBIA (T55D26S, Abmart, Shanghai, China), and anti-NF-κB p65 (80979-1-RR, Proteintech, Wuhan, China). Horseradish peroxidase (HRP)-conjugated secondary antibodies included anti-mouse IgG (LF101, EpiZyme, Shanghai, China) and anti-rabbit IgG (LF102, EpiZyme, Shanghai, China). All primary and secondary antibodies were used at the dilutions listed in Supplementary Table S2.

### 2.10. Enzyme-Linked Immunosorbent Assay

Cell culture supernatants were collected from each group, and cytokine levels were determined using the following porcine-specific ELISA kits, according to the manufacturers’ instructions: SEKP-0009 Porcine TNF-α ELISA Kit, SEKP-0001 Porcine IL-1β ELISA Kit, SEKP-0005 Porcine IL-8 ELISA Kit, SEKP-0019 Porcine MCP-1 ELISA Kit, and SEKP-0004 Porcine IL-6 ELISA Kit (Solarbio, Beijing, China). Before detection, all kit components and samples were allowed to equilibrate to room temperature, and standard solutions were prepared as described in the kit manuals. Supernatants were used directly or appropriately diluted with the provided assay buffer to ensure that all readings fell within the linear range of the standard curve. For the assay, 100 μL of each standard or sample was added to the respective wells and incubated for 60 min at 37 °C, followed by five washes with wash buffer provided in the kit. Subsequently, 100 μL of enzyme-labeled reagent was added to each well, and the plates were incubated for another 60 min at 37 °C, followed by five additional washes. Then, 100 μL of chromogenic substrate was added to each well, and the plates were incubated for 15 min at 37 °C in the dark. Finally, 50 μL of stop solution was added to terminate the reaction, and the absorbance was read at 450 nm with a reference wavelength of 630 nm using a microplate reader. The final absorbance values were calculated as the difference between the readings at 450 nm and 630 nm (A_450_–A_630_) to correct for background interference. Cytokine levels in each sample were quantified based on the corresponding standard curve. The detection ranges and sensitivities were as follows: TNF-α, 7.81–500 pg/mL and 4 pg/mL; IL-1β, 31.25–2000 pg/mL and 15 pg/mL; IL-8, 31.25–2000 pg/mL and 15 pg/mL; MCP-1, 6.25–400 pg/mL and 3 pg/mL; and IL-6, 62.5–4000 pg/mL and 31.25 pg/mL. The intra- and inter-assay coefficients of variation for all kits were <10%, and the total reaction time was approximately 2.33 h. All samples were assayed in duplicate, and the results are expressed as mean values.

### 2.11. Transcriptomic Analysis

Porcine ovarian granulosa cells were divided into three groups: a control group (Control), an OTA group, and an OTA+RES group, with five biological replicates per group, resulting in a total of 15 samples. Total RNA was extracted as described above.

After quality control of the raw sequencing data, fastp [32] was employed to remove adapter sequences and low-quality reads, and metrics such as base quality and GC content were calculated. The resulting clean reads were aligned to the reference genome using STAR to generate sorted BAM files, and alignment statistics were computed. Transcript assembly and quantification were subsequently performed using StringTie [33] to calculate FPKM/TPM values. Meanwhile, featureCounts was used to generate a gene-level read count matrix. Differential expression analysis was conducted using DESeq2 in R Studio (2026.01.1), and the expression patterns were visualized using principal component analysis (PCA), hierarchical clustering heatmaps, and volcano plots. Furthermore, functional enrichment analysis of the differentially expressed genes was performed to elucidate their potential biological significance.

### 2.12. Statistical Analysis

All experiments were performed in at least three independent biological replicates, and the results were expressed as mean ± standard deviation (mean ± SD). Data collation and statistical analyses were performed using GraphPad Prism 10.1.2 software (GraphPad Software, San Diego, CA, USA). The independent-sample t-test was used for comparisons between two independent groups when data satisfied normality and homogeneity of variance; the non-parametric Mann–Whitney U test was used when these assumptions were violated. For comparisons among three or more groups, one-way ANOVA followed by appropriate post hoc multiple comparisons was applied for data meeting normality and homogeneity of variance. The non-parametric Kruskal–Wallis test followed by Dunn’s multiple comparisons test was used when data failed to meet these assumptions [34,35]. Statistical significance in the figures was denoted as follows: “ns” = not significant; * *p* < 0.05; ** *p* < 0.01; *** *p* < 0.001. Different lowercase letters above the bars indicate statistically significant differences between groups (*p* < 0.05).

## 3. Results

### 3.1. RES Pretreatment Alleviates OTA-Induced Damage to Granulosa Cells

**Figure 1:**
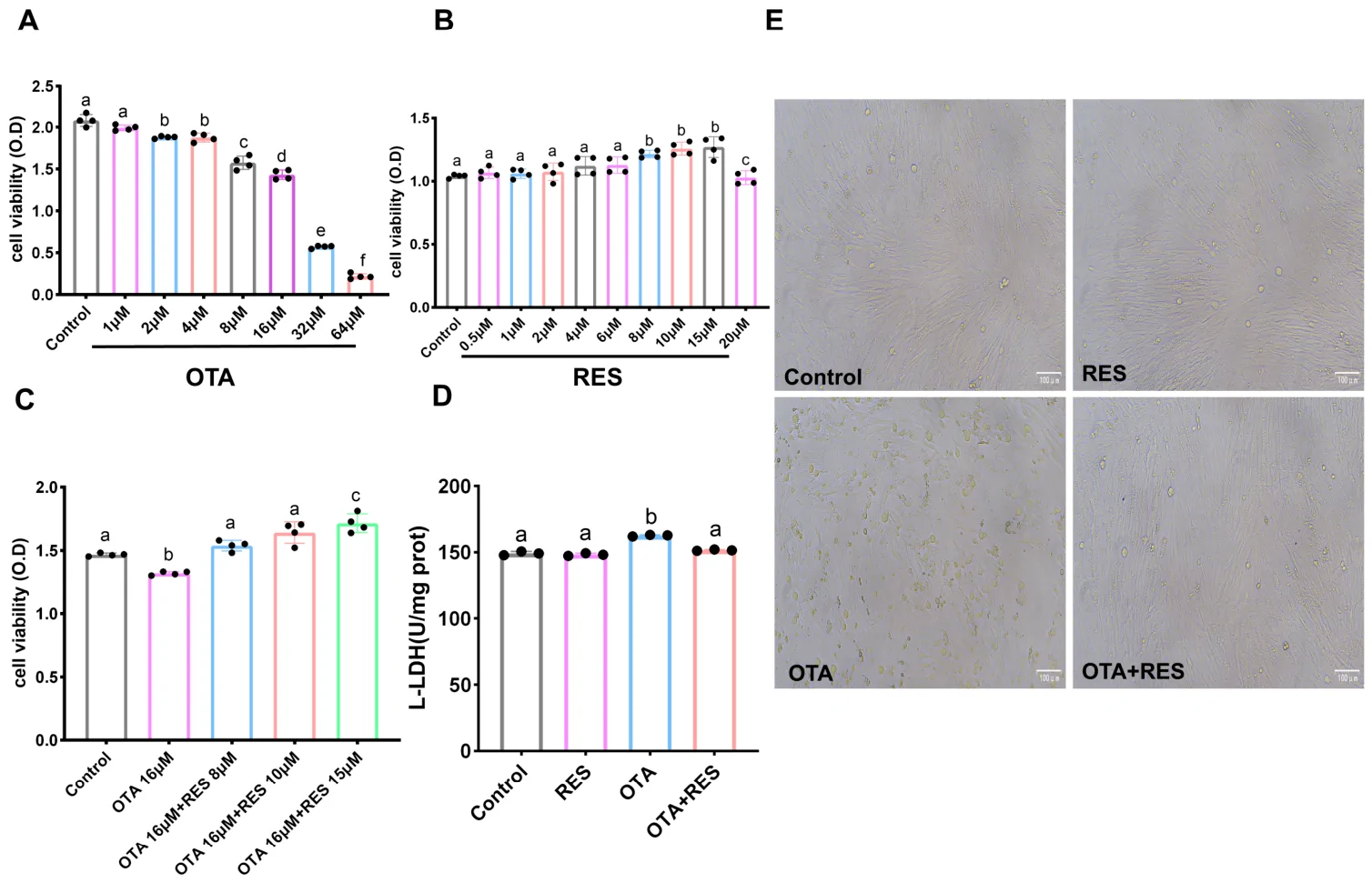
Effect of RES on OTA-induced cell viability and cell damage in porcine ovarian granulosa cells. (**A**) Cell viability after treatment with increasing concentrations of OTA. (**B**) Cell viability after treatment with increasing concentrations of RES. (**C**) Cell viability after co-treatment with OTA and increasing concentrations of RES. (**D**) L-LDH levels in each group. (**E**) Morphological observation of porcine ovarian granulosa cells in each group. Scale bar: 100 µm. Lowercase letters above bars denote statistically significant differences. Groups sharing at least one common lowercase letter are not significantly different, whereas groups with no shared lowercase letters indicate significant differences (*p* < 0.05). Data are presented as mean ± standard deviation (SD) from three independent experiments (*n* = 3).

To determine the appropriate concentrations of OTA for establishing a cellular injury model and the optimal concentration of RES for rescue, cell viability was assessed using the CCK-8 assay after treatment with various concentrations of OTA (0, 1, 2, 4, 8, 16, 32, 64 μM) or RES (0, 0.5, 1, 2, 4, 8, 10, 15, 20 μM) for 24 h (Figure 1A–C). As shown in Figure 1A, the viability of porcine ovarian granulosa cells decreased in a dose-dependent manner with increasing concentrations of OTA. Compared with the control group, OTA at 1 μM had no significant effect on cell viability, whereas treatment with 4–64 μM OTA for 24 h resulted in a significant, dose-dependent reduction in viability. Additionally, 8 μM OTA caused only a mild reduction in cell viability, and this weak cytotoxic phenotype was insufficient to support stable mechanistic investigations; in comparison, 16 μM OTA produced stable and reproducible cell damage without excessive cell death, making it the optimal concentration. Therefore, 16 μM OTA was selected to establish the porcine ovarian granulosa cell injury model. Regarding RES, concentrations ranging from 0.5 to 6 μM did not significantly affect cell viability, while treatment with 8–15 μM RES for 24 h significantly increased cell viability. However, the effect slightly declined at 20 µM (Figure 1B). Furthermore, as illustrated in Figure 1C, cell viability in the OTA-alone group was significantly decreased compared with the control group. Co-treatment with 8, 10 or 15 μM RES markedly alleviated OTA-induced cytotoxicity in porcine ovarian granulosa cells. Notably, cell viability in the 15 μM RES group was significantly higher than that in the 10 μM RES group, and the value was also significantly greater than that of the control group. These findings indicate that 15 μM RES exerts the optimal rescue effect, and this concentration was therefore selected for subsequent rescue experiments. Consistent with the cell viability results, the LDH release assay showed that OTA significantly increased LDH release compared with the control, while co-treatment with RES significantly decreased LDH release compared with the OTA-alone group and restored it to the control level, further confirming the protective effect of RES against OTA-induced cell damage (Figure 1D). Morphological observation under a microscope (Figure 1E) revealed that cells in the control group exhibited a regular shape and good adherence. Cells in the RES-alone group showed no obvious morphological differences from the control group. In contrast, cells in the OTA-treated group displayed marked shrinkage, rounding, and floating, indicating cellular damage. Notably, the co-treatment group (OTA+RES) exhibited a significantly improved morphology compared with the OTA-alone group, suggesting that RES effectively alleviates OTA-induced morphological damage.

### 3.2. RES Alleviates OTA-Induced Oxidative Stress in Porcine Ovarian Granulosa Cells

**Figure 2:**
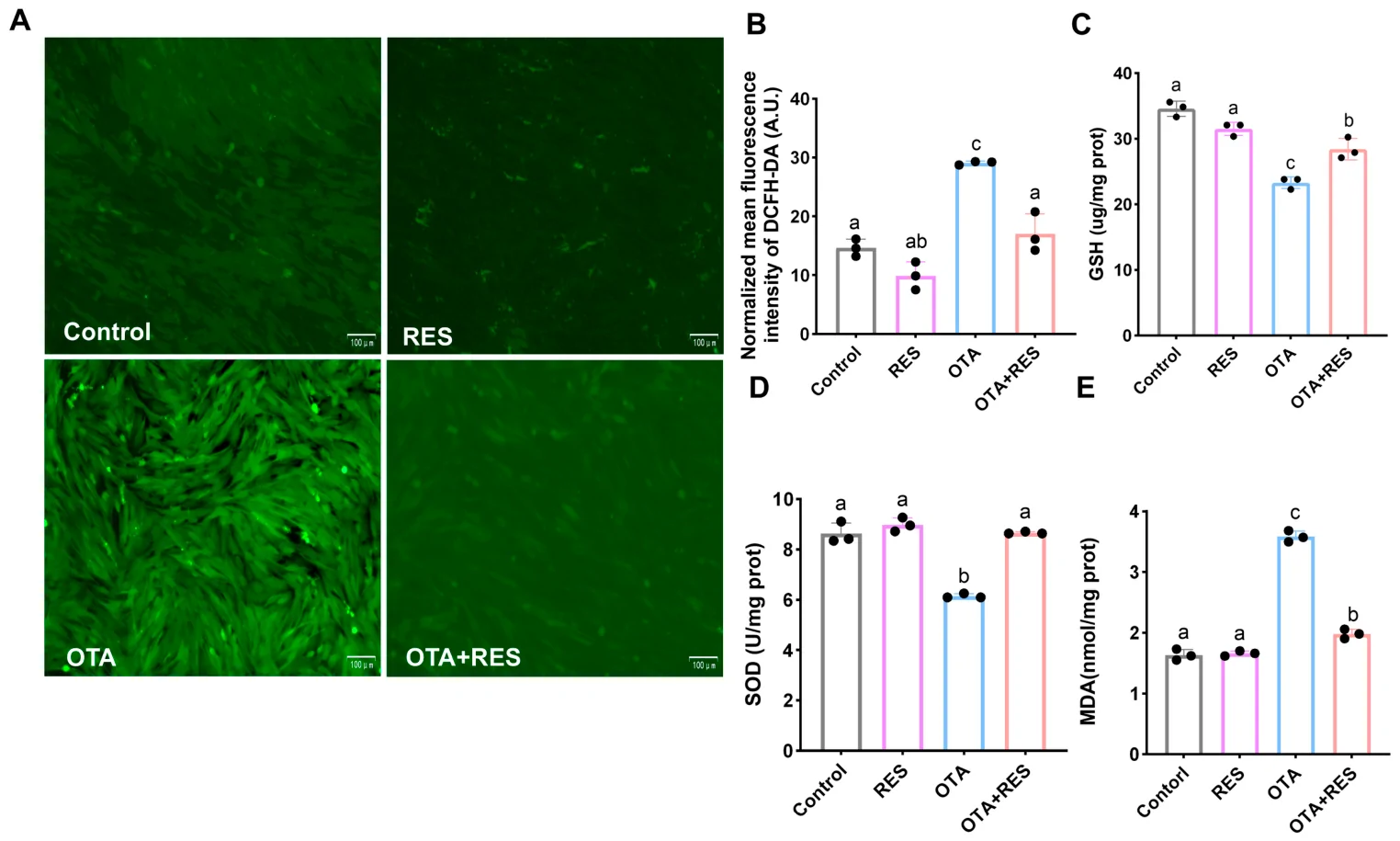
Effects of RES on OTA-induced reactive oxygen species levels and antioxidant indicators in porcine ovarian granulosa cells. (**A**) Representative fluorescence staining images of reactive oxygen species (ROS) in porcine ovarian granulosa cells of each group (control, untreated control group; OTA, OTA-treated group; RES, RES-treated group; OTA+RES, group co-treatment with RES and OTA). Scale bar: 100 µm. (**B**) Quantitative analysis of ROS levels in each group. (**C**) Reduced glutathione (GSH) levels in each group. (**D**) Superoxide dismutase (SOD) activity in each group. (**E**) Malondialdehyde (MDA) levels in each group. Lowercase letters above bars denote statistically significant differences. Groups sharing at least one common lowercase letter are not significantly different, whereas groups with no shared lowercase letters indicate significant differences (*p* < 0.05). Data are presented as mean ± standard deviation (SD) from three independent experiments (*n* = 3).

To investigate the regulatory effect of RES on OTA-induced oxidative stress, intracellular reactive oxygen species (ROS) levels and oxidative stress-related indicators, including malondialdehyde (MDA), superoxide dismutase (SOD), and reduced glutathione (GSH), were measured. As shown in Figure 2A,B, intracellular ROS levels were significantly increased in the OTA-treated group relative to the control group. The ROS level was markedly reduced in the OTA+RES co-treatment group compared with the OTA group, and no significant difference was observed when compared with the control group. This indicates that RES co-treatment can significantly reverse OTA-induced ROS accumulation, restoring the ROS level to a level comparable to that of the control group. In addition, OTA treatment led to a significant increase in MDA content and a marked decrease in SOD activity and GSH content. In contrast, the RES rescue group significantly ameliorated these abnormal changes (Figure 2C–E). Meanwhile, SOD activity in the OTA+RES group returned to the control level, whereas GSH and MDA contents remained slightly different from the control, demonstrating that OTA- induced oxidative stress-related indicators was partially reversed. Collectively, these results indicate that RES effectively alleviates OTA-induced toxicity in porcine ovarian granulosa cells by attenuating oxidative stress damage.

### 3.3. RES Alleviates OTA-Induced Apoptosis in Porcine Ovarian Granulosa Cells

**Figure 3:**
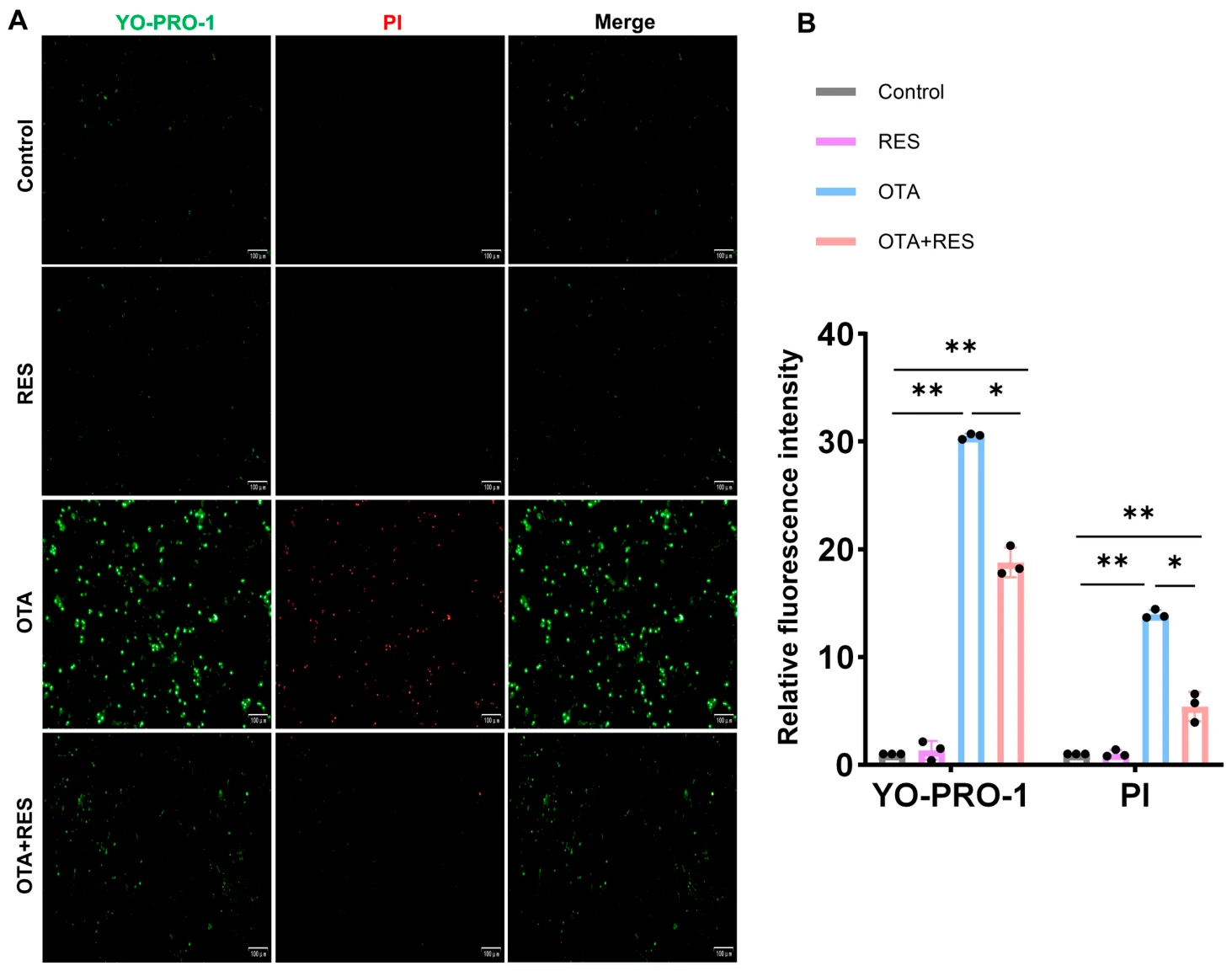
Fluorescence images of apoptosis detected by YO-PRO-1/PI double staining in porcine ovarian granulosa cells of each group. (**A**) YO-PRO-1 labels early apoptotic cells (green), and propidium iodide (PI) labels late apoptotic/necrotic cells (red). Merge represents the overlayed images. Scale bar: 100 µm. (**B**) Relative fluorescence intensity of YO-PRO-1 and PI in each group. Data are presented as mean ± standard deviation (SD) from three independent experiments (*n* = 3). ns, not significant; * *p* < 0.05; ** *p* < 0.01.

To investigate the protective effect of RES (RES) against OTA-induced apoptosis, YO-PRO-1 and propidium iodide (PI) double staining was used to evaluate the impact of OTA treatment on cell apoptosis. YO-PRO-1-positive cells were identified as early apoptotic cells, and PI-positive cells as late apoptotic or necrotic cells. As shown in (Figure 3A,B), in the control group and the RES-alone group, very few YO-PRO-1- and PI-positive cells were observed, with only minimal green and red fluorescence detected. In contrast, the OTA-treated group exhibited a significant increase in the number of YO-PRO-1- and PI-positive cells, accompanied by markedly enhanced green and red fluorescence intensity. However, the OTA+RES group still exhibited significantly higher fluorescence intensities than the control group, suggesting that although RES effectively attenuated OTA induced apoptosis, it failed to the basal level of the control group. These results demonstrate that RES effectively alleviates OTA-induced apoptosis in porcine ovarian granulosa cells.

### 3.4. RES Alleviates OTA-Induced Transcriptomic Alterations in Porcine Ovarian Granulosa Cells

**Figure 4:**
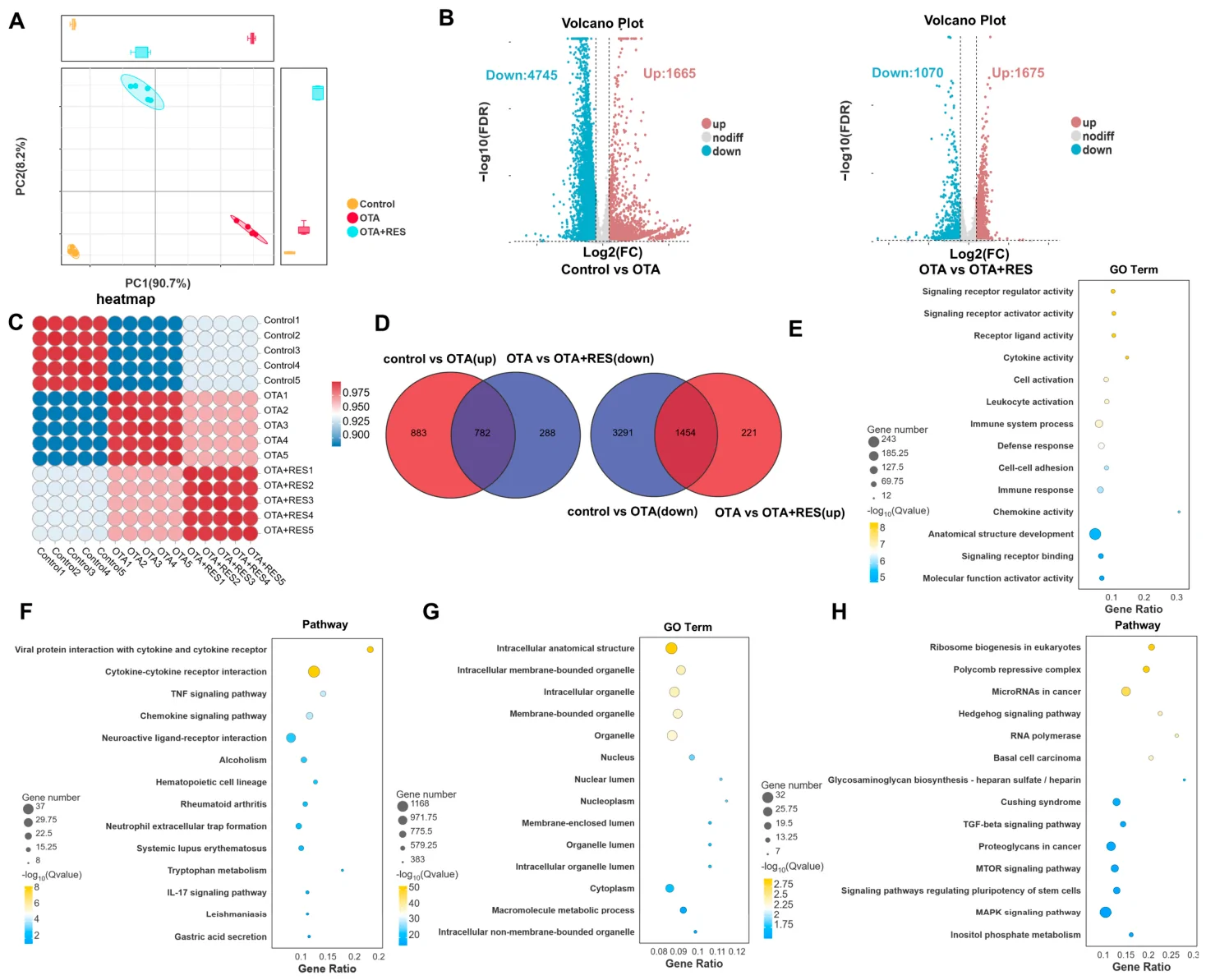
RES ameliorates OTA-induced transcriptomic changes in porcine ovarian granulosa cells. (**A**) Principal component analysis (PCA) plot of different groups. The first principal component (PC1) explains 90.7% of the variance, and the second principal component (PC2) explains 2.8% of the variance. (**B**) Volcano plot of differentially expressed genes (DEGs) between groups. Red dots indicate significantly upregulated genes, blue dots indicate significantly downregulated genes, and grey dots indicate genes with no significant difference. (**C**) Sample-to-sample correlation heatmap of the RNA-seq data. (**D**) Venn diagram of DEGs among different groups. (**E**,**F**) GO terms and KEGG pathways enriched for DEGs that were upregulated in the OTA group but downregulated in the OTA+RES group. (**G**,**H**) GO terms and KEGG pathways enriched for DEGs that were downregulated in the OTA group but upregulated in the OTA+RES group.

To elucidate the functional consequences and mechanistic basis of OTA cytotoxicity in porcine ovarian granulosa cells, we conducted RNA-seq analysis on three groups: Control, OTA-treated, and OTA+RES co-treatment samples. Principal component analysis (PCA) was then applied to evaluate global transcriptional divergence across experimental cohorts and the reproducibility within each group. As illustrated in Figure 4A, the PCA plot revealed a distinct segregation among Control, OTA-treated, and OTA+RES co-treatment samples. Specifically, the control and OTA-treated groups were distinctly separated along the PC1 axis, suggesting that OTA treatment induced significant transcriptomic alterations in porcine ovarian granulosa cells. Notably, the OTA+RES group was located between the control and OTA-treated groups and was closer to the control group, indicating that RES partially reversed the OTA-induced transcriptomic abnormalities.

The differential expression analysis results (Figure 4B) showed that compared with the control group, a total of 6410 DEGs were identified in the OTA-treated group, of which 1665 were upregulated and 4745 were downregulated. Compared with the OTA-treated group, 2745 DEGs were identified in the OTA+RES group, including 1675 upregulated and 1070 downregulated genes. To assess the similarity of global transcriptomic profiles among different treatment groups, sample-to-sample correlation analysis was performed and visualized as a correlation heatmap (Figure 4C). Biological replicates within each group exhibited high consistency, indicating excellent reproducibility of the RNA-seq data. The OTA-treated samples displayed a transcriptomic profile distinct from that of the Control group, whereas the OTA+RES group exhibited greater similarity to the Control group, suggesting that RES partially restored the OTA-induced transcriptional alterations. To further elucidate the regulatory effect of RES on OTA-induced gene expression abnormalities, an intersection analysis was performed between the DEGs from the OTA group and those from the OTA+RES group. As shown in Figure 4D, 782 DEGs that were upregulated in the OTA group but downregulated in the OTA+RES group were identified, along with 1454 DEGs that were downregulated in the OTA group but upregulated in the OTA+RES group.

To explore the molecular mechanisms underlying OTA-induced transcriptomic changes, GO functional enrichment and KEGG [36] pathway enrichment analyses were performed on the upregulated and downregulated differentially expressed genes (DEGs) in the OTA-treated group. As shown in Figure 4E, GO enrichment analysis of the upregulated genes following OTA exposure revealed that they were primarily enriched in functional categories related to signal transduction regulation, immune response, and cell activation. KEGG pathway enrichment analysis (Figure 4E,F) further demonstrated that the upregulated genes were mainly enriched in cytokine signal transduction, inflammatory immune responses, and infection-related disease pathways. GO enrichment analysis of the downregulated genes following OTA exposure (Figure 4G,H) showed that they were predominantly enriched in cellular components (nucleus, cytoplasm, and membrane-bound organelles) and metabolic processes (organic substance metabolism, primary metabolism, and nitrogen compound metabolism), suggesting that OTA exposure broadly suppresses the expression of genes involved in cellular structure and basal metabolic functions. KEGG pathway enrichment analysis (Figure 4F) further revealed that the downregulated genes were mainly enriched in transcription and translation processes (ribosome biogenesis, RNA polymerase), multiple signal transduction pathways (MAPK, Hedgehog, TGF-β, Hippo, HIF-1, mTOR), as well as pathways related to development and differentiation.

### 3.5. RES Exerts Anti-Inflammatory Protection by Ameliorating OTA-Induced NF-κB/HIF Pathway Alterations

**Figure 5:**
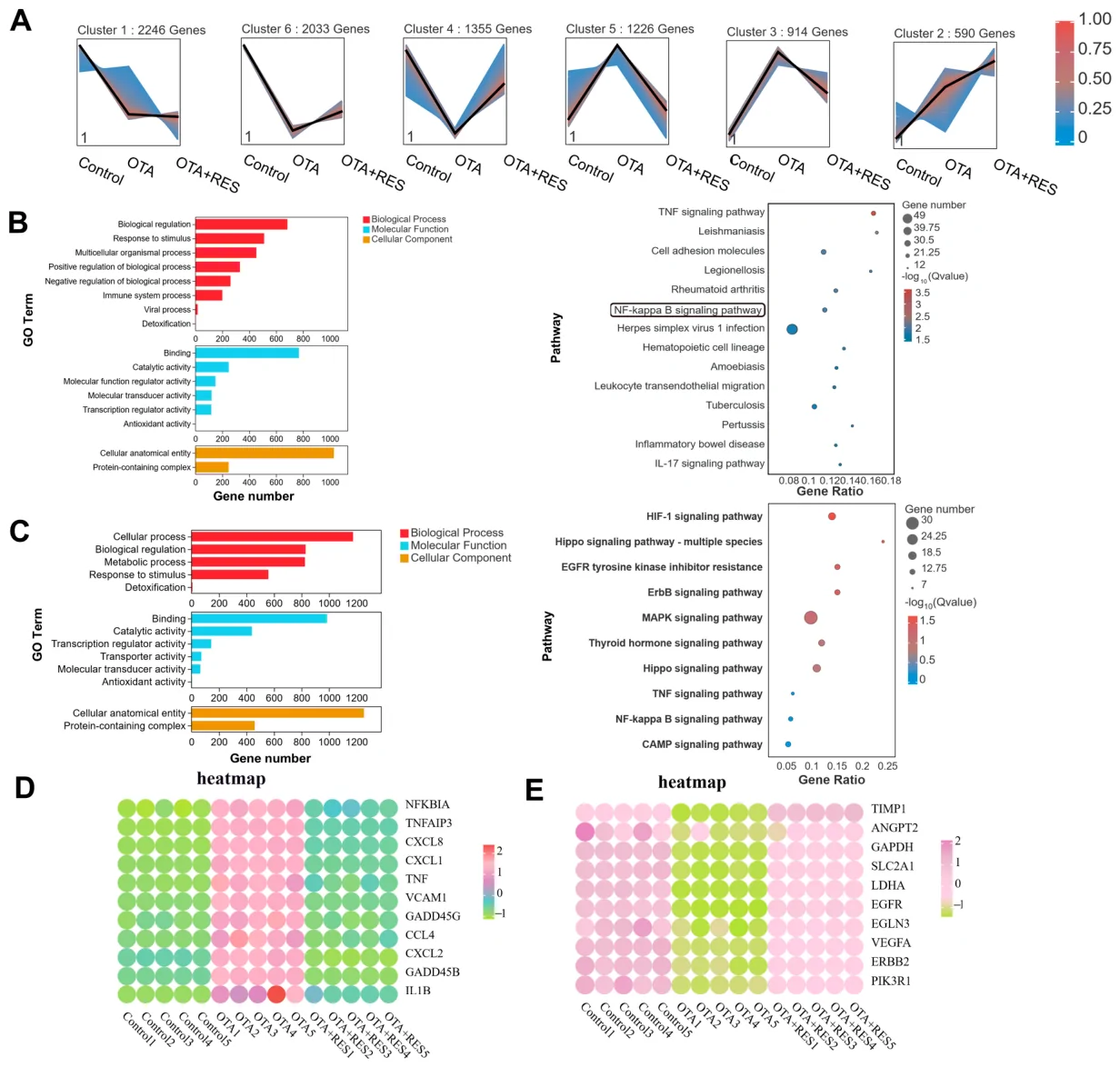
RES exerts anti-inflammatory protective effects by ameliorating the OTA-induced NF-κB/HIF pathway. (**A**) Trend analysis of DEGs across the three groups (Control, OTA, OTA+RES). (**B**) GO terms and KEGG pathways enriched for DEGs in cluster 5 (upregulated in the OTA group and downregulated in the OTA+RES group). (**C**) GO terms and KEGG pathways enriched for DEGs in cluster 4 (downregulated in the OTA group and upregulated in the OTA+RES group). (**D**,**E**) Heatmap of DEGs involved in the NF-κB pathway (from cluster 5) and the HIF-1 signaling pathway (from cluster 4).

To further investigate the regulatory mechanisms of RES on gene expression, trend analysis was performed on the differentially expressed genes (DEGs) from the control group, OTA group, and OTA+RES group. A total of six gene clusters were identified (Figure 5A). Among these, Cluster 4 (1355 genes) and Cluster 5 (1226 genes) exhibited the most pronounced differential expression trends across the three groups and were therefore considered the core targets for subsequent functional enrichment analysis.

Functional enrichment analysis was first performed on the intersecting genes of Cluster 5. The results showed that OTA exposure significantly activated a functional network centered on immune response and stress response. At the molecular function level, genes related to binding activity, catalytic activity, and transcriptional regulatory activity were most significantly enriched (Figure 5B). At the pathway level, the TNF/NF-κB/IL-17 inflammatory signaling axis, cell adhesion molecules, and the leukocyte transendothelial migration pathway were markedly activated. Additionally, multiple signal transduction pathways, including MAPK, HIF-1, and Hippo, were also involved (Figure 5B). Subsequently, functional enrichment analysis was performed on Cluster 4. The results revealed that OTA exposure significantly suppressed a functional network centered on basal metabolism and cellular homeostasis maintenance. At the biological process level, genes related to cellular processes, biological regulation, metabolic processes, and stress response were most significantly enriched. At the molecular function level, genes associated with binding activity, catalytic activity, transcriptional regulatory activity, and antioxidant activity were markedly downregulated. At the cellular component level, the expression of genes related to cellular anatomical entities and protein-containing complexes was also suppressed (Figure 5C). At the pathway level, multiple signaling pathways associated with metabolism and cellular homeostasis maintenance exhibited a downward trend (Figure 5C). These results suggest that the genes in this cluster may play a potential regulatory role in RES-mediated alleviation of OTA-induced toxicity in porcine ovarian granulosa cells.

To further identify the core regulatory pathways, heatmap analysis was performed on the NF-κB pathway gene set from Cluster 5 and the HIF-1 pathway gene set from Cluster 4. The gene expression profiles showed that pro-inflammatory target genes related to the NF-κB signaling pathway were significantly upregulated in the OTA-treated group, whereas their expression was markedly downregulated in the RES rescue group to levels comparable to those in the control group (Figure 5D). In contrast, the core genes of the HIF-1 signaling pathway, involved in glycolytic metabolic reprogramming, angiogenesis, oxygen sensing regulation, and upstream signal activation, were significantly downregulated in the OTA group, but their expression was substantially restored in the RES rescue group, exhibiting an opposite expression trend to that observed in the OTA group (Figure 5E).

In summary, the above results demonstrate that RES exerts a protective effect against OTA-induced reproductive toxicity in porcine ovarian granulosa cells by reversing the aberrant activation of the NF-κB pathway and the suppressed state of the HIF-1 pathway, thereby counteracting the stress-related transcriptional reprogramming induced by OTA.

### 3.6. RES Co-Treatment Alleviates OTA-Induced Inflammation in Ovarian Granulosa Cells

**Figure 6:**
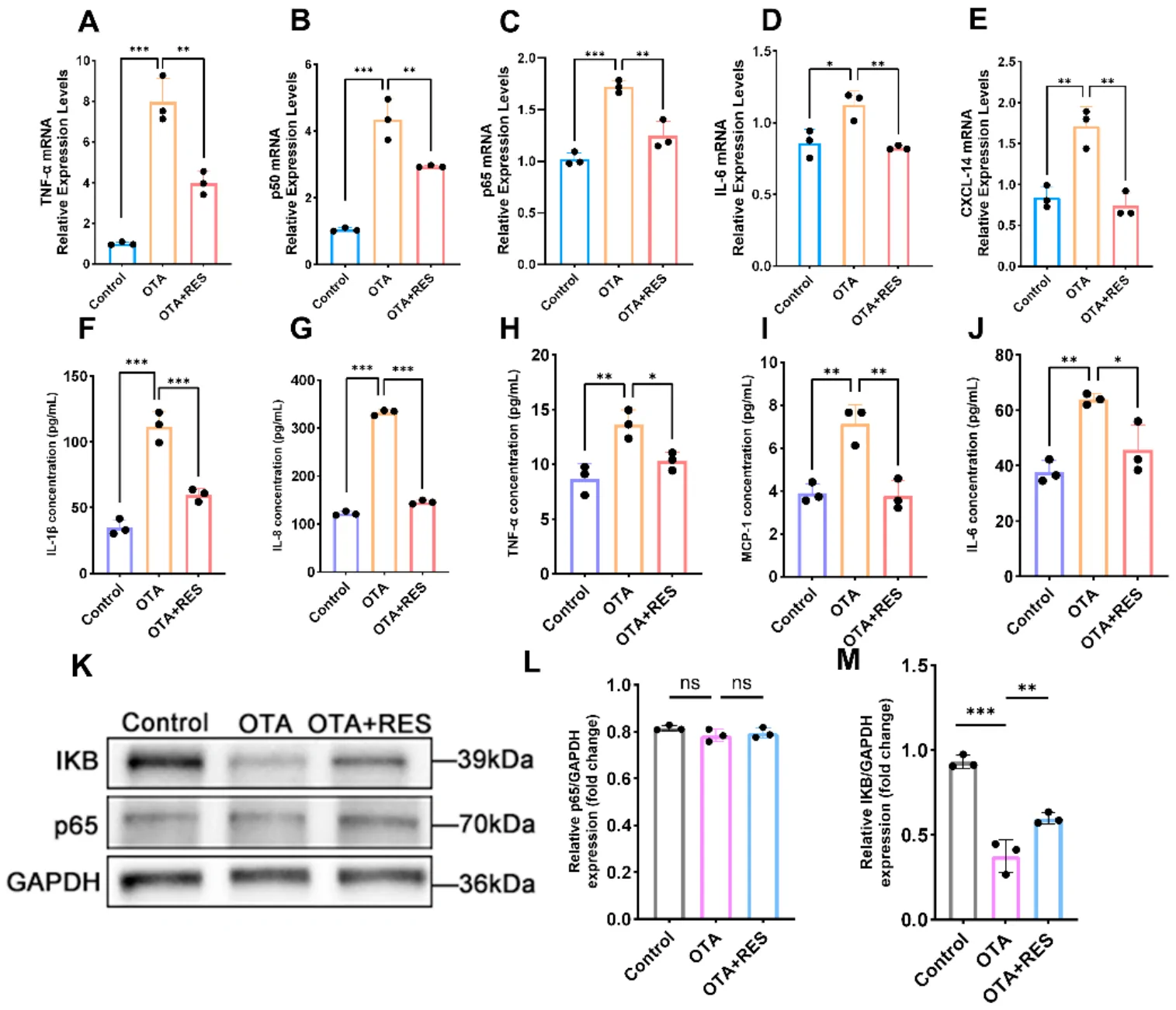
RES co-treatment alleviates OTA-induced inflammation in ovarian granulosa cells. (**A**) Relative mRNA expression level of TNF-α. (**B**) Relative mRNA expression level of p50. (**C**) Relative mRNA expression level of p65. (**D**) Relative mRNA expression level of IL-6. (**E**) Relative mRNA expression level of CXCL-4. (**F**) Concentrations of IL-1β in the culture supernatants were measured by ELISA. (**G**) Concentrations of IL-8 in the culture supernatants were measured by ELISA. (**H**) Concentrations of TNF-α in the culture supernatants were measured by ELISA. (**I**) Concentrations of MCP-1 in the culture supernatants were measured by ELISA. (**J**) Concentrations of IL-6 in the culture supernatants were measured by ELISA. (**K**) Protein expression levels of IκB and p65 were detected by Western blotting, with GAPDH used as a loading control. (**L**) Quantitative analysis of p65 protein expression levels among the three groups. (**M**) Quantitative analysis of IκB protein expression levels among the three groups. Data are presented as mean ± standard deviation (SD) from three independent experiments (*n* = 3). ns, not significant; * *p* < 0.05; ** *p* < 0.01; *** *p* < 0.001.

To evaluate the effect of RES on the inflammatory response of porcine ovarian granulosa cells following OTA exposure, the expression levels of inflammatory factors were measured in three differently treated groups. The results showed that OTA treatment significantly upregulated the mRNA expression levels of *TNF-α*, *p50*, *p65*, *IL-6*, and *CXCL-14*, whereas RES rescue group alleviated these changes (Figure 6A–E). After measuring the levels of inflammatory factors in the culture supernatant, it was found that OTA treatment significantly promoted the secretion of IL-1β, IL-8, TNF-α, MCP-1, and IL-6. In contrast, the protein levels of these inflammatory factors were markedly reduced in the RES rescue group (Figure 6F–J). To further analyze the effect of OTA treatment on NF-κB signaling pathway activation and the protective role of RES, Western blotting was performed to detect the expression changes in p65 and IκBα in porcine ovarian granulosa cells under different treatment conditions. The results showed that p65 protein levels remained largely unchanged among the three groups. Regarding IκBα protein levels, compared with the control group, the OTA-treated group exhibited a marked reduction in IκBα expression, whereas the RES rescue group showed higher IκBα levels than the OTA-treated group, approaching the levels observed in the control group (Figure 6K–M). These data indicated that OTA activates the NF-κB inflammatory cascade by triggering IκBα degradation, whereas RES stabilizes IκBα protein to suppress excessive NF-κB activation and alleviate cellular inflammatory injury. Taken together, these results support that RES alleviates OTA-triggered inflammatory responses in a robust manner.

## 4. Discussion

As a widely distributed mycotoxin, OTA poses a considerable threat to human and animal health through its nephrotoxic, hepatotoxic, and reproductive toxic effects. In the present study, we employed porcine ovarian granulosa cells as an in vitro model and combined phenotypic assays, transcriptomic sequencing, and molecular biological validation to systematically investigate the mechanisms underlying OTA-induced cytotoxicity. In parallel, we identified that RES, a naturally occurring polyphenol, alleviates OTA-provoked cell damage, primarily by counteracting oxidative stress, which in turn suppresses NF-κB-mediated inflammatory responses and apoptosis, ultimately preserving granulosa cell viability.

Initial dose–response experiments established 16 μM OTA as the optimal concentration for constructing a cellular injury model in porcine granulosa cells, while 15 μM RES was found to markedly improve cell viability and morphology [22,37]. Granulosa cells are somatic cells that surround oocytes and play an essential role in follicular development, hormone secretion, and oocyte maturation. At appropriate levels, intracellular reactive oxygen species (ROS) participate in physiological processes such as folliculogenesis, ovulation, and corpus luteum formation. However, excessive ROS accumulation induces oxidative stress, which impairs granulosa cell proliferation, promotes apoptosis, and triggers follicular atresia, ultimately compromising sow reproductive performance [28]. Our data revealed that OTA exposure dramatically elevates intracellular ROS levels, accompanied by depletion of endogenous antioxidants including GSH and SOD, alongside increased malondialdehyde (MDA) production—a hallmark of lipid peroxidation—indicating a robust oxidative stress response. These observations align with earlier reports showing that OTA exposure leads to ROS accumulation in oocytes, disrupting spindle assembly and chromosome alignment, thereby causing meiotic arrest and aneuploidy [38]. Moreover, other mycotoxins such as aflatoxins, zearalenone, and deoxynivalenol have also been shown to elicit oxidative stress in various cell types [39,40]. Notably RES supplementation significantly lowered ROS levels and restored endogenous antioxidant enzyme activities, corroborating its well-established function as both a radical scavenger and an inducer of antioxidative enzyme [41,42]. Interestingly, a previous study by Cano-Sancho et al. found that resveratrol failed to alleviate OTA-induced cytotoxicity in Caco-2 intestinal epithelial cells, and even enhanced the cytotoxic effects of OTA at high concentrations. In particular, co-exposure to 80 μM OTA and 100 μM resveratrol resulted in increased cytotoxicity, but ROS production did not increase accordingly [43]. We speculate that these discrepancies may be attributed to differences in cell type and treatment concentration. Collectively, in the specific experimental context of porcine ovarian granulosa cells, RES can alleviate OTA-mediated oxidative stress, thereby promoting cell growth.

Oxidative stress arising from excessive ROS accumulation is widely recognized as a critical upstream driver of apoptosis [44]. Upon exposure to adverse environmental stimuli—such as nutrient deprivation, radiation, or toxic chemicals [45,46]—cellular autophagic and apoptotic pathways are often triggered. In our experiments, YO-PRO-1/PI dual staining of OTA-exposed granulosa cells yielded a marked increase in fluorescence signal, confirming the induction of apoptosis. This finding is consistent with prior investigations in porcine intestinal epithelial IPEC-J2 cells and oocytes, where OTA was shown to impair mitochondrial function and activate apoptotic cascades via ROS generation, with these effects being significantly rescued by antioxidants such as Mito-TEMPO and melatonin [38,47]. As expected, RES treatment substantially reduced the proportion of positive-staining cells, indicating its capacity to suppress apoptosis. The anti-apoptotic effect of RES, mediated through mitigation of oxidative stress, has been proposed in multiple experimental models [48]. For instance, in cardiomyocytes, RES exerts cardioprotective effects by activating FOXO3a, which curtails ROS accumulation and inhibits apoptotic signaling [49]. Likewise, RES attenuates fungal-induced allergic inflammation and apoptosis via the Akt/mTOR pathway, thereby relieving endoplasmic reticulum stress [50,51]. Taken together, these lines of evidence strongly support that RES effectively counters apoptosis driven by oxidative stress.

We next sought to gain deeper mechanistic insights into OTA-induced cytotoxicity in porcine ovarian granulosa cells. Trend clustering analysis of differentially expressed genes from RNA-seq, combined with KEGG pathway enrichment, revealed that genes upregulated upon OTA exposure were predominantly associated with the NF-κB inflammatory signaling pathway, whereas downregulated genes were significantly enriched in the HIF-1 signaling pathway. Notably, RES treatment reversed these expression patterns, yielding a trend opposite to that observed in the OTA group. Previous reports have shown that OTA induces glomerular injury through activation of the ERK/NF-κB axis [52], and also promotes hepatic inflammation and gut microbiota dysbiosis by facilitating Gram-negative bacterial outgrowth and NF-κB activation [53]. In agreement with these findings, our Western blot data confirmed a marked reduction in IκB, the inhibitory subunit of the NF-κB pathway, following OTA exposure. Moreover, qRT-PCR and ELISA analyses further corroborated the transcriptomic results, revealing substantial increases in both mRNA and protein levels of pro-inflammatory cytokines including TNF-α, IL-6, IL-1β, IL-8, and MCP-1, collectively pointing to robust inflammatory pathway activation.

Multiple lines of evidence have also linked RES to inflammation modulation. For instance, RES attenuates SIRT1/NF-κB-regulated inflammation after spinal cord injury in rats, thereby diminishing neuronal loss and lesion size [54]. In a murine model of ankylosing spondylitis, RES restores intestinal barrier integrity and reshapes gut microbial composition through suppression of inflammatory responses and the TLR4/NF-κB/NLRP3 cascade [55]. These observations are in line with our own data, wherein RES administration effectively rescued the hyperactivation of the NF-κB pathway triggered by OTA. Our results thus highlight the important anti-inflammatory function of RES in porcine ovarian granulosa cells.

Nevertheless, several limitations of the present study should be acknowledged. We have systematically evaluated the direct biological effects of resveratrol at the cellular-phenotype level using the resveratrol-only group, including cell viability, oxidative status and apoptosis. However, due to constraints in sample preparation and sequencing budget, the resveratrol-only group was not included in our transcriptome sequencing. Therefore, based on the available transcriptomic data, we can only decipher the protective-rescue effect of resveratrol against OTA-induced toxic damage, and cannot interpret the independent transcriptional alterations directly triggered by resveratrol itself in normal granulosa cells. Follow-up transcriptomic experiments with the resveratrol-only group will help to fully uncover its intrinsic transcriptional regulatory functions.

In conclusion, our findings demonstrate that OTA exposure induces oxidative stress in porcine ovarian granulosa cells, which subsequently activates NF-κB-mediated inflammation, culminating in decreased cell viability and apoptosis. Given its antioxidant and anti-inflammatory properties, RES represents a promising candidate as a pharmaceutical or feed additive to counteract mycotoxin-induced toxicity. However, our current study is limited to only porcine ovarian granulosa cells. Therefore, whether the conclusions drawn in this work exhibit cell-type specificity requires further controlled experiments using additional generic cell models in future investigations. Meanwhile, in subsequent studies, we will focus on improving the experimental design by adding a RES-only group for transcriptome analysis to further investigate the intrinsic biological effects of RES itself.

## Data Availability

All data generated or analyzed during this study are included in this published article. The RNA-seq raw data have been deposited in the National Genomics Data Center (NGDC) under BioProject PRJCA065493 and are available at https://ngdc.cncb.ac.cn/gsub/submit/bioproject/PRJCA065493/overview (accessed on 14 October 2026).

## Supplementary Materials

The following supporting information can be downloaded at: https://www.mdpi.com/article/10.3390/antiox15101339/s1, Table S1: Primers for qPCR used in this work. Table S2: Details of antibodies.

## Abbreviations

Nonstandard Abbreviations: Ochratoxin A, OTA; RES, RES; Cell Counting Kit-8, CCK-8; Hypoxia-inducible factor-1, HIF-1; Nuclear factor kappa-B, NF-κB; Nuclear factor erythroid 2-related factor 2, NRF2; Enzyme-linked immunosorbent assay, ELISA; Reactive oxygen species, ROS; Glutathione, GSH; Lactate dehydrogenase, LDH; Malondialdehyde, MDA; Superoxide dismutase, SOD; C-X-C motif chemokine ligand 14, CXCL-14; Interleukin, IL.
