# Supplementary material for: Mechanism of Resveratrol in Alleviating Ochratoxin A-Induced Damage to Porcine Ovarian Granulosa Cells
Source: Antioxidants (Basel). 2026 Oct 16;15(10):1339. doi: 10.3390/antiox15101339 (PMC13701169; doi:10.3390/antiox15101339)
Supplement: Supplementary file 1 [file antioxidants-15-01339-s001.zip › antioxidants-4533915-supplementary.pdf]

Supplementary Results

RES Co-Treatment Alleviates OTA-Induced Inflammation in Ovarian Granulosa Cells

To further evaluate the effect of RES on the inflammatory response of porcine ovarian granulosa cells following OTA exposure, the expression levels of inflammatory factors were measured across four differently treated groups: Control, OTA-treated, RES-treated, and OTA+RES co-treatment samples. The results showed that OTA treatment significantly upregulated the mRNA expression levels of TNF-α, p50, p65, IL-6, and CXCL-14, whereas the RES rescue group alleviated these changes. Meanwhile, in the RES-alone treatment group, the mRNA level of TNF-α was significantly increased compared with the control group, whereas no significant changes were observed in the expression of other inflammatory factors (Supplementary Fig. 6A-E). After measuring the protein levels of inflammatory factors in the culture supernatant, it was found that OTA treatment significantly promoted the secretion of IL-1β, IL-8, TNF-α, IL-6, and MCP-1. In contrast, the protein levels of these inflammatory factors were markedly reduced in the RES rescue group (Supplementary Fig. 6F-J). In addition, there were no significant differences in the protein levels of these inflammatory factors in the RES-alone group relative to the control group. These data indicated that RES co-treatment effectively suppresses OTA-induced inflammatory cytokine production and secretion in ovarian granulosa cells. Taken together, these supplementary results further support that RES alleviates OTA-triggered inflammatory responses in a robust manner.

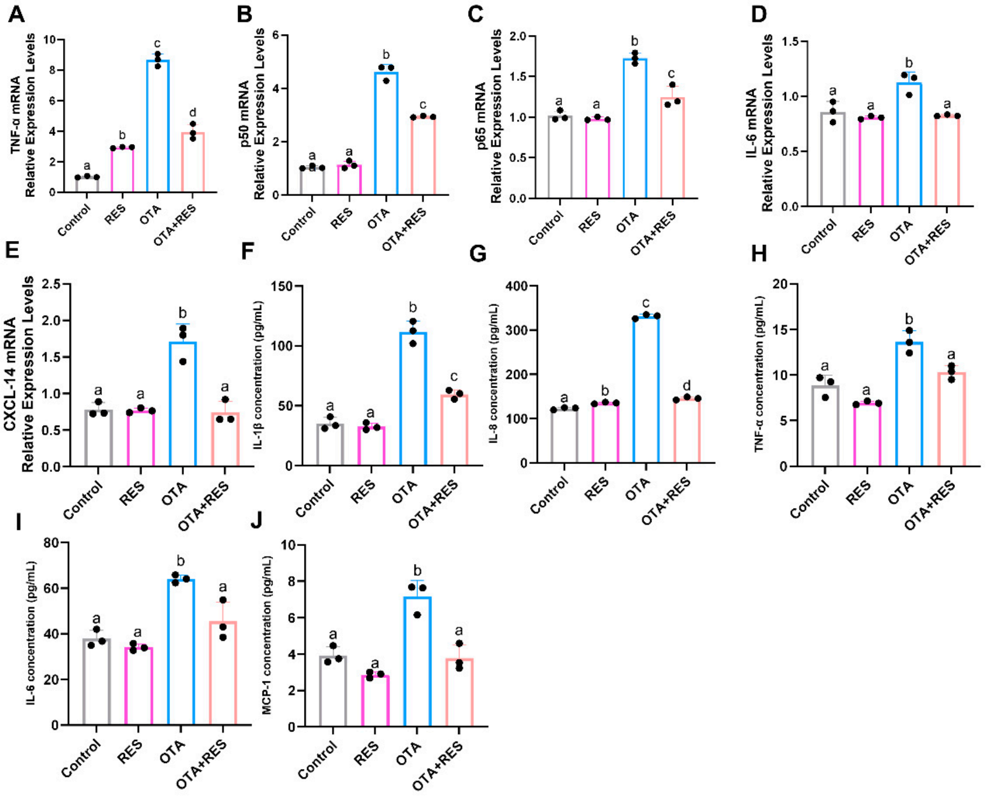

Supplementary Figure S1. RES co-treatment alleviates OTA-induced inflammation in ovarian granulosa cells. A Relative mRNA expression level of TNF-α. B Relative mRNA expression level of p50. C Relative mRNA expression level of p65. D Relative mRNA expression level of IL-6. E Relative mRNA expression level of CXCL-14. F Concentrations of IL-1β in the culture supernatants were measured by ELISA. G Concentrations of IL-8 in the culture supernatants were measured by ELISA. H Concentrations of TNF-α in the culture supernatants were measured by ELISA. I Concentrations of IL-6 in the culture

supernatants were measured by ELISA. J Concentrations of MCP-1 in the culture supernatants were measured by ELISA. Data are presented as mean ± standard deviation (SD) from three independent experiments (n = 3). Different lowercase letters (a, b, c, d) indicate significant differences between groups ($p < 0.05$).

Supplementary Table S1. Primers for qPCR used in this work

Gene	Primer Sequences（5'-3'）	Annealing Temperature	NCBI Gene ID
β-actin	F: TGCGGGACATCAAGGAGAAG<br>R: AGTTGAAGGTGGTCTCGTGG	57.34°C	414396
TNF-α	F: TGGCCCAAGGACTCAGATCAT<br>R: ATTGGCATACCCACTCTGCC	58.90°C	397086
IL-6	F: GGCTGCTTCTGGTGATGGCTA<br>R: TTGCCTCAGGGTCTGGATCAGT	63.79°C	399500
P50	F:CCCATGTAGACAGCACCACCTATGAT<br>R:ACAGAGGCTCAAAGTTCTCCACCA	63.40°C	751869
P65	F: CATGCGCTTCCGCTACAAG<br>R: GGTCCCGCTTCTTTACACAC	57.40°C	100135665
CXCL-14	F: AGGGACCCAAGATCCGCTAC<br>R: CTATTCTTCATAGACCCTGCGCT	59.60°C	494467

Supplementary Table S2. Details of Antibodies.

Antibody	SOURCE	Host Species	Catalog Code	Dilution
				WB
Anti-Mouse IgG	EpiZyme, China	Goat	LF101	1:5000
Anti- Rabbit IgG	EpiZyme, China	Goat	LF102	1:5000
Anti- GAPDH	EpiZyme, China	Mouse	LF205	1:5000
Anti- NFKBIA	Abmart, China	Rabbit	T55D26S	1:1000
Anti- NF-κB p65	Proteintech	Rabbit	80979-1-RR	1:1000
